# Serum tumour M2‐pyruvate kinase as a biomarker for diagnosis and prognosis of early‐stage non–small cell lung cancer

**DOI:** 10.1111/jcmm.16762

**Published:** 2021-07-13

**Authors:** Chunhua Xu, Wei Liu, Li Li, Yuchao Wang, Qi Yuan

**Affiliations:** ^1^ Department of Respiratory Medicine Nanjing Chest Hospital Nanjing China; ^2^ Affiliated Nanjing Brain Hospital Nanjing Medical University Nanjing China

**Keywords:** biomarker, diagnosis, non, prognosis, small cell lung cance, tumour M2‐pyruvate kinase

## Abstract

Tumour M2‐pyruvate kinase (TUM2‐PK) is up‐regulated in many human cancers. This study was to evaluate the clinical value of serum TUM2‐PK in early‐stage non–small cell lung cancer (NSCLC) patients. A total of 162 consecutive early‐stage NSCLC patients were enrolled and followed up after tumour resection. Serum TUM2‐PK level was detected by enzyme‐linked immunosorbent assay (ELISA) in NSCLC patients, 50 benign pulmonary disease patients and 102 healthy controls. The TUM2‐PK level in NSCLC patients was higher than that of healthy controls (*P* < .001) and benign pulmonary disease patients (*P* < .001). A threshold of 30 U/mL could be used to diagnose early‐stage NSCLC with 71.6% sensitivity and 98.0% specificity. The 5‐year overall survival rate in patients with high TUM2‐PK level was lower than that of patients with low TUM2‐PK level (*P* = .009). Multivariable Cox regression showed that high TUM2‐PK level was an independent risk factor for overall survival (HR = 2.595, 95% CI: 1.231‐5.474, *P* = .012). High serum TUM2‐PK level could be a potential biomarker for diagnosis and prognosis of early‐stage NSCLC patients.

## 1 INTRODUCTION

Non–small cell lung cancer (NSCLC) is a malignant tumour with a high incidence, high mortality and poor prognosis.^1^ Surgical resection is the main treatment for stage I‐IIIA NSCLC. Routine adjuvant chemotherapy with platinum can improve the prognosis of patients with I‐III NSCLC.^2^, ^3^, ^4^ However, there is still controversy about the adjuvant therapy of stage I NSCLC during the current period. About 40%‐50% of patients with early NSCLC still have local recurrence or/and distant metastasis after surgery, and the 5‐year overall survival rate is 60%‐90%.^5^, ^6^ TNM stage is the main means to assess the degree of involvement of lung cancer lesions.^7^ However, the prognosis of patients with similar stage characteristics may still have differences.^8^ Therefore, in early NSCLC, screening high‐risk patients who may have recurrence or metastasis, and guiding more active systemic and local treatment on this basis may be of great significance to prolong the overall survival of patients.

Tumor M2 pyruvate kinase is a key enzyme in glycolytic, which plays an important role in tumour metabolism. The expression level of TUM2‐PK was increased in many tumour patients, and it was closely related to the pathogenesis of tumour. It is highly expressed in tumour cells, such as gastrointestinal cancer, ovarian cancer and other tumours.^9^, ^10^, ^11^, ^12^, ^13^, ^14^ Previous study indicated that blood TUM2‐PK levels in lung cancer patients were higher than those in the healthy controls.^15^ Schneider et al applied TUM2‐PK to differentiate SCLC patients, which can improve the diagnosis of SCLC.^16^ However, the value of serum TUM2‐PK in the diagnosis and prognosis of early NSCLC is still unclear.

The aim of this study was to explore the application value of TUM2‐PK in the early diagnosis of lung cancer, to study the relationship between serum TUM2‐PK level and clinicopathological features, and to evaluate the prognostic value of TUM2‐PK in NSCLC patients, so as to provide a new biomarker for the diagnosis, screening and individualized treatment of early NSCLC.

## 2 METHODS

### 2.1 Patients

This study was approved by the Ethics Committee of Nanjing Chest Hospital and informed consent of patients. From September 2015 to May 2018, radical resection and systematic lymph node dissection of lung cancer was performed, and the diagnosis was confirmed by pathology, cytology and histopathology. According to the eighth edition of the Union for International Cancer Control, tumour‐node‐metastasis (TNM) classification of lung cancer was performed to identify the early NSCLC. Exclusion criteria include the following: incomplete imaging data; unable to determine the clinical stage; chest computed tomography (CT), abdominal B ultrasound, head magnetic resonance imaging (MRI), bone scan, etc were used to diagnose metastatic cancer or complicated with other malignant tumours; patients with previous tumour history or pre‐operative adjuvant treatment such as radiotherapy and chemotherapy; patients who could not follow the study protocol to complete the follow‐up. There were 162 cases of lung cancer, 50 cases of benign lung disease and 102 cases of healthy control. The blood samples of normal people were from healthy people in Nanjing Chest Hospital. The selection criteria were people whose physical examination, blood, liver and kidney function and imaging results were normal. Patients' clinical information and serum sample test results were collected by doctors who did not directly participate in the diagnosis and treatment and detection process and entered into the database.

### 2.2 Determination of TUM2‐PK

After admission, 2ml of fasting venous blood, anti‐coagulation of EDTA‐K2, centrifugation and freezing at −80°C were taken before measurement. The concentration of TUM2‐PK was determined by ELISA. All kits were purchased from ScheBo Tech, Germany. The assay was performed on automatic biochemical analyser in strict accordance with the instructions of the kit.

### 2.3 Follow‐up

The patients were followed up every six months after operation. Chest CT, abdominal B ultrasound, head MRI and bone scan were performed. The time and location of tumour recurrence or metastasis, the time and cause of death were recorded. Follow‐up data were obtained by outpatient review, telephone and communication.

### 2.4 Statistical analysis

SPSS 22.0 software was used to analyse data. After the homogeneity test of square error, the *t* test was used to compare the data of two groups of independent samples in accordance with the homogeneity of variance. Analysis of variance was used to compare multiple groups of independent samples. To evaluate the diagnostic and prognostic abilities of the TUM2‐PK, area under the receiver operating characteristic (ROC) curve (AUC) values were determined for the indicated patient groups, and the specificity and sensitivity were calculated for biomarkers’ cut‐off levels. The survival time was from operation date to death time or the last follow‐up time. Kaplan‐Meier method was applied to describe the survival curve, and log‐rank test was applied to compare the differences between groups. Cox proportional hazard regression model was applied to evaluate the influence of TUM2‐PK level on post‐operative survival. *P* < .05 was the difference statistically significant.

## 3 RESULTS

### 3.1 Clinical characteristics

A total of 162 patients with early NSCLC diagnosed pathologically were included, including 95 females and 67 males. The average age was 58.8 years. There were 103 cases in stage I, 59 cases in stage II, 126 cases were adenocarcinoma, 36 cases were squamous cell carcinoma, 38 cases were well differentiation, 60 cases were moderate differentiation and 64 cases were poor differentiation. There were 50 cases of benign pulmonary diseases, including 20 cases of male and 30 cases of female, with an average age of 53.5 years. The pathological diagnosis was inflammation in 20 cases, tuberculosis in 10 cases, hamartoma in 8 cases, sclerosing hemangioma in 8 cases, adenomatous hyperplasia in 2 cases and cyst in 2 cases. There were 102 healthy control group, 58 males, 44 males, mean age 55.4 years. There was no significant difference in age and gender among three groups.

### 3.2 The value of serum TUM2‐PK level in the diagnosis of early NSCLC

**FIGURE 1:**
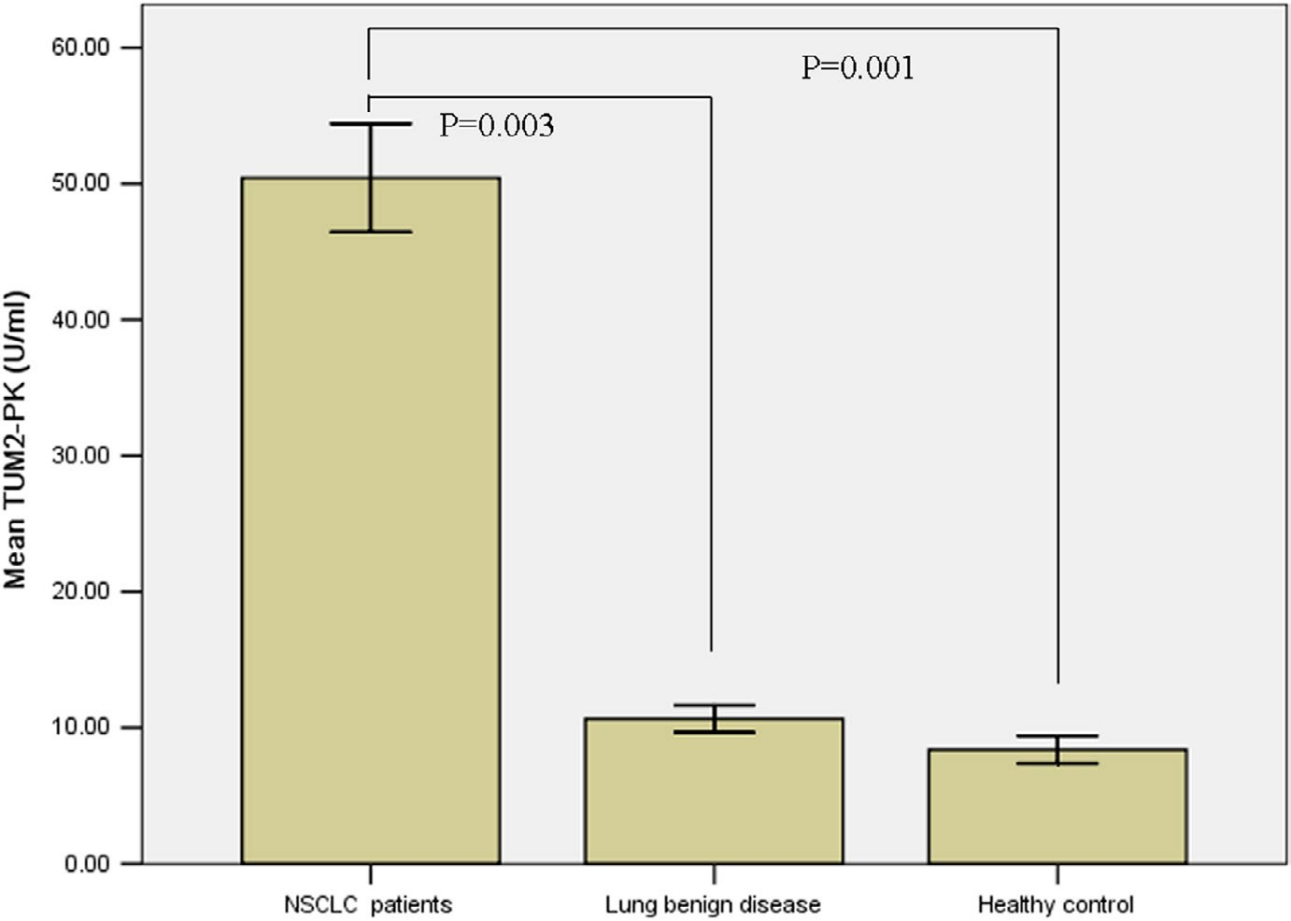
Serum levels of TUM2‐PK in of NSCLC patients, lung benign disease patients and healthy control

Compared with benign lung disease and the control, TUM2‐PK level of NSCLC patient (50.4 ± 12.5 U/mL) was higher than that in benign lung disease (*P* < .05) and the control (*P* < .05). There was no difference between benign lung disease and the control (10.7 ± 1.8 U/mL vs 8.4 ± 1.8 U/mL) (Figure 1).

**FIGURE 2:**
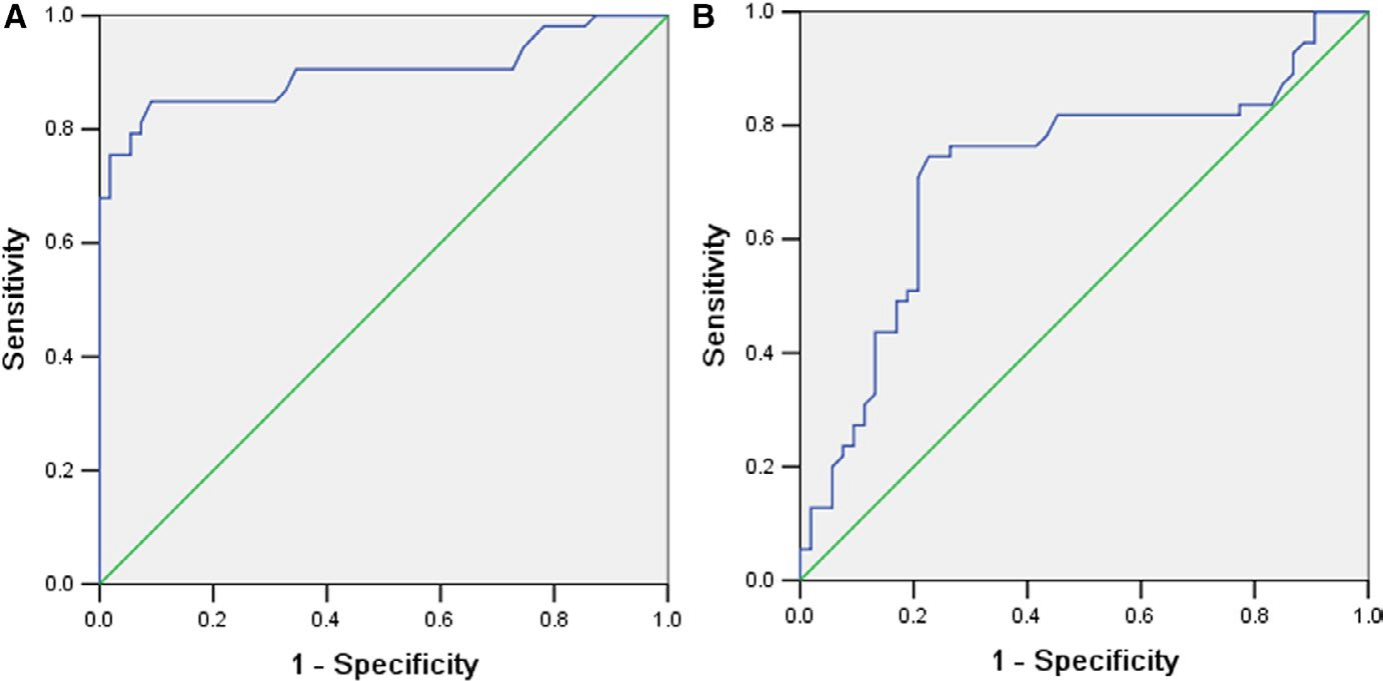
The ROC curve of serum TUM2‐PK for the diagnosis of lung cancer (A); the ROC curve of serum TUM2‐PK level for predicting post‐operative outcomes within 5 years after surgery survival (B)

Receiver operating characteristic curve was drawn with the concentration of TUM2‐PK in lung cancer and the control. The area under curve (AUC) of TUM2‐PK in the diagnosis of NSCLC was 0.901 (95% CI: 0.835‐0.966) (Figure 2A). The cut‐off value afforded by the ROC analysis for TUM2‐PK was 30 U/ml. The sensitivity and specificity of TUM2‐PK in detecting NSCLC were 71.6% and 98.0%, respectively.

### 3.3 Association between TUM2‐PK level and clinicopathological characteristics

The serum level of TUM2‐PK was associated with tumour differentiation (*P* = .044), lymph node metastasis (*P* = .014), TNM stage (*P* = .029) and post‐operative mortality (*P* = .028) (Table 1). The serum TUM2‐PK level was increased in patients with stage II, poor differentiation and lymph node metastasis. No correlation was found between TUM2‐PK level and pathological type, age, gender, smoking status and recurrence or metastasis of NSCLC within five years after operation (*P* > .05).

**TABLE 1:** Comparison of the serum TUM2‐PK level among different clinicopathological characteristics

Characteristics	N	TUM 2‐PK (U/mL)	*P*
Age(years)			.465
<60	92	48.7 ± 13.5	
≥60	70	51.7 ± 11.8	
Gender			.227
Male	67	48.1 ± 12.2	
Female	95	52.9 ± 12.7	
Smoking history (years)			.235
<20	94	47.7 ± 12.4	
≥20	68	52.5 ± 12.4	
Pathological type			.863
SCC	36	50.1 ± 13.8	
ADC	126	50.8 ± 11.4	
Differentiation			.044
Moderate‐well	98	45.8 ± 12.4	
Poor	64	53.8 ± 11.6	
Stage			.029
I	103	46.2 ± 12.8	
II	59	54.7 ± 10.8	
Lymph node metastasis			.014
No	134	44.3 ± 13.2	
Yes	28	54.5 ± 10.3	
Recurrence/metastasis			.383
No	127	48.6 ± 12.0	
Yes	35	52.1 ± 12.9	
Chemotherapy			.056
No	120	51.2 ± 9.8	
Yes	42	50.7 ± 11.2	
Survival information			.028
Alive	139	46.9 ± 13.6	
Dead	23	55.1 ± 9.2	

Abbreviations: ADC, adenocarcinoma SCC; squamous cell carcinoma.

### 3.4 Relationship between serum TUM2‐PK level and prognosis of early NSCLC

The total follow‐up time was 5 years, and the median follow‐up time was 47.8 months. During the follow‐up period, 23 cases (14.2%) died of tumour, and the 5‐year survival rate was 85.8%; 35 cases (21.6%) had recurrence or metastasis, including 8 cases of local recurrence (4.9%) and 27 cases of distant metastasis (16.7%).

During the follow‐up period, the level of serum TUM2‐PK in dead patients was significantly higher than that in survivors (55.1 ± 9.2 U/mL vs 46.9 ± 13.6 U/mL, *P* = .042) (Table 1). The serum TUM2‐PK level of patients with early NSCLC who died within 5 years after operation was used to make ROC curve. The AUC was 0.720 (95% CI: 0.619‐0.821) (Figure 2B). According to ROC curve, when the cut‐off value was 46 U/mL, the sensitivity and specificity were 72.7% and 69.6% to predict the poor prognosis during the follow‐up period.

According to the TUM2‐PK level, lung cancer patients were divided into high‐level TUM2‐PK (TUM2‐PK ≥46 U/mL) and low‐level TUM2‐PK group (TUM2‐PK <46 U/mL) (Table 2). Compared with low serum TUM2‐PK level group, NSCLC patients with high serum TUM2‐PK level were more common in stage II, poor differentiation and lymph node metastasis patients (*P* < .05). The number of patients who died also increased significantly (Table 3).

**TABLE 2:** Association of potential predictors with overall survival after surgery for patients with early‐stage NSCLC

Variable	Overall survival	
	HR (95% CI)	*P*
Univariate analysis		
Age (<60 vs ≥60)	1.008 (0.965‐1.054)	.708
Gender (Male vs Female)	1.007 (0.968‐1.046)	.740
Pathological type (SCC vs ADC)	1.042 (0.778‐1.393)	.786
Differentiation (Well‐moderate vs Poor)	1.432 (0.670‐3.062)	.354
Lymph node metastases (Yes vs No)	1.010 (0.999‐1.021)	.064
TNM stage (I vs II)	1.158 (1.102‐1.218)	.001
Chemotherapy (Yes vs No)	0.865 (0.613‐1.220)	.407
TUM2‐PK (High vs Low)	5.331 (1.833‐15.507)	.002
Multivariate analysis		
Age (<60 vs ≥60)	1.004 (0.993‐1.015)	.524
Gender (Male vs Female)	1.044 (0.552‐1.975)	.894
Pathological type (SCC vs ADC)	0.997 (0.958‐1.036)	.862
Differentiation (Well‐moderate vs Poor)	0.736 (0.269‐2.012)	.551
Lymph node metastases (Yes vs No)	1.316 (0.536‐3.232)	.549
TNM stage (I vs II)	2.964 (1.642‐7.926)	.016
Chemotherapy (Yes vs No)	1.123 (0.558‐2.259)	.745
TUM2‐PK (High vs Low)	2.595 (1.231‐5.474)	.012

Abbreviations: ADC, adenocarcinoma.CI, confidence interval; HR, hazard ratio; SCC, squamous cell carcinoma.

**TABLE 3:** The baseline and follow‐up of patients with different serum TUM2‐PK level

Characteristics	TUM2‐PK <46 U/mL (n = 99)	TUM2‐PK ≥46 U/mL (n = 63)	*P*
Age(years)			.195
<60	52 (52.5%)	40 (63.5%)	
≥60	47 (47.5%)	23 (36.5%)	
Gender			.141
Male	36 (36.4%)	31 (49.2%)	
Female	63 (63.6%)	32 (50.8%)	
Smoking history (years)			.628
<20	59 (59.6%)	35 (55.6%)	
≥20	40 (40.4%)	28 (44.4%)	
Pathological type			.847
SCC	23 (23.2%)	13 (20.6%)	
ADC	76 (76.8%)	50 (79.4%)	
Differentiation			.022
Moderate‐well	67 (67.7%)	31 (49.2%)	
Poor	32 (32.3%)	32 (50.8%)	
Stage			.008
I	71 (71.7%)	32 (50.8%)	
II	28 (28.3%)	31 (49.2%)	
Lymph node metastasis			.035
No	87 (87.9%)	47 (74.6%)	
Yes	12 (12.1%)	16 (25.4%)	
Recurrence/metastasis			.117
No	82 (82.8%)	45 (71.4%)	
Yes	17 (17.2%)	18 (28.6%)	
Chemotherapy			.382
No	72 (72.7%)	48 (76.2%)	
Yes	27 (27.3%)	15 (23.8%)	
Survival information			.001
Alive	94 (94.9%)	45 (71.4%)	
Dead	5 (5.1%)	18 (28.6%)	

Abbreviations: ADC, adenocarcinoma; SCC, squamous cell carcinoma.

**FIGURE 3:**
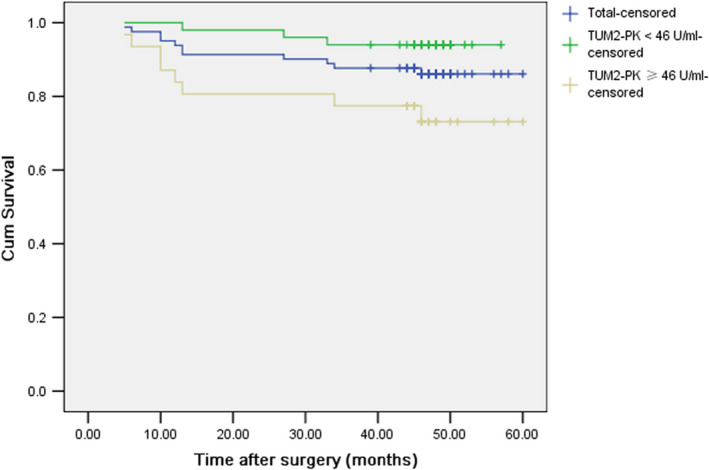
Overall survival curves and 5‐year survival rate of patients with high level of TUM2‐PK and low level of TUM2‐PK

Kaplan‐Meier survival curves showed that the 5‐year overall survival rate was lower in patients with high serum TUM2‐PK than in patients with low serum TUM2‐PK (71.4% vs 94.9%) (Figure 3). The patients with high serum TUM2‐PK had earlier post‐operative mortality (*P* < .05), 80% of the death events in the low serum TUM2‐PK group occurred between 12 and 18 months after surgery, while those in the high serum TUM2‐PK group occurred between 6 and 12 months after surgery, suggesting that the two groups of patients may need different follow‐up strategies.

Univariate Cox survival analysis showed that the risk of death in patients with high TUM2‐PK level was 5.331 times higher than that in patients with low TUM2‐PK level (HR = 5.331, 95% CI: 1.833‐15.507, *P* = .002). Further exclusion of sex, age, smoking history, lymph node metastasis, stage and other factors, multivariate Cox proportional hazard model suggested that high serum level of TUM2‐PK was an independent risk factor for post‐operative mortality (HR = 2.595, 95% CI 1.231‐5.474, *P* = .012) (Table 2).

## 4 DISCUSSION

At present, the unsatisfactory situation of lung cancer treatment is that most patients still have recurrence and metastasis after treatment. Even after surgical resection, subclinical metastases still exist in patients after surgery. Even if adjuvant chemotherapy is used, it is still difficult to avoid tumour recurrence due to the inhibition of apoptosis process of tumour cells. Imaging examination and bronchoscopy have important value in the diagnosis and recurrence detection of lung cancer. They are commonly used clinical medical methods, but they are not easy to use in large‐scale screening. A large number of studies and prevention data have confirmed that early diagnosis and treatment is the most effective way to prevent and reduce mortality. The level of tumour markers has a good correlation with the occurrence, development and recurrence of tumours. Therefore, it has been a focus of attention to find tumour markers that can be used for early diagnosis and prognosis.

Tumor M2 pyruvate kinase is a newly discovered tumour marker in recent years. To explore its role in tumour diagnosis, curative effect evaluation and prognosis evaluation has become a hot research at home and abroad.^17^, ^18^ The expression of TUM2‐PK in serum of normal people was stable at low level and increased significantly in tumour state. The serum level of TUM2‐PK in lung cancer patients, patients with benign lung diseases and healthy controls was studied. It was found that the serum TUM2‐PK level in NSCLC patients was significantly higher than that in the controls and benign lung diseases patients. It suggested that TUM2‐PK might play an important role in the occurrence and development of lung cancer. When the threshold value of TUM2‐PK was set at 30 U/mL, the sensitivity and specificity of serum TUM2‐PK were 71.6% and 98.0%, respectively. It indicated that TUM2‐PK had important application value in the early diagnosis of lung cancer and might become a biomarker for diagnosis and screening of lung cancer.

The serum TUM2‐PK level in patients with stage II lung cancer was higher than that in patients with stage I lung cancer, and the expression level of TUM2‐PK in patients with lymph node metastasis was higher than that in patients without lymph node metastasis, suggesting that the level of TUM2‐PK may be related to the local invasion of early lung cancer. The results are consistent with those of foreign studies.^19^, ^20^ The reason may be related to the progress of tumour, the release of more TUM2‐PK in the process of tumour cell necrosis or metastasis. In this study, there was no significant correlation between serum TUM2‐PK level and pathological type, age, gender and smoking history in early NSCLC patients.

In the present study, our results suggest that TUM2‐PK may be a biomarker for predicting prognosis in patients with early NSCLC. The 5‐year overall survival rate of patients with high serum TUM2‐PK was lower than that of patients with low serum TUM2‐PK. To further rule out the potential influence of age, sex, smoking history, tumour differentiation and other factors on the outcome, it was indicated that pre‐operative high serum TUM2‐PK level was an independent risk factor for post‐operative death in patients with early NSCLC. Therefore, pre‐operative serum TUM2‐PK may be of great significance in screening high‐risk patients with poor post‐operative prognosis of early lung cancer. Compared with other invasive and molecular pathology methods, the evaluation of serum markers has the advantages of simplicity, rapidity and reproducibility and may be helpful in understanding patient risk stratification before surgery, to guide the choice of treatment plan and strengthen adjuvant therapy. In addition, patients with different TUM2‐PK levels also had differences in the time of death after surgery, suggesting that patients with low TUM2‐PK levels should emphasize follow‐up within 18 months, while those with high TUM2‐PK levels should be followed up regularly. Future studies could further confirm whether high‐risk patients can indeed benefit from treatment guided by the serum TUM2‐PK detection.

As the study population is early NSCLC patients, but the adjuvant treatment and intensive treatment of early patients are still controversial, post‐operative adjuvant treatment may be partially different. Since only a few patients in this study received post‐operative adjuvant therapy, the analysis of the relationship between post‐operative adjuvant therapy and prognosis of patients may be biased. The application of these findings to other patients will require more research and exploration.

The main mechanism of TUM2‐PK in lung cancer may be related to its role in tumour metabolism. The increase of dimer TUM2‐PK content is often accompanied by the occurrence of tumour, while the decrease or even disappearance of tetramer TUM2‐PK content. In solid tumours (such as lung cancer) with sufficient oxygen supply, glutamic acid provides a large amount of pyruvate. The pyruvate produced in glutamic acid and glycolysis is used to synthesize lactic acid, glutamic acid and fatty acid, so as to release the hydrogen produced by glyceraldehyde 3‐phosphate dehydrogenase in the process of glycolysis, so as to ensure the energy supply in tumour cells.

To sum up, the detection of serum TUM2‐PK level may have important clinical significance for predicting the prognosis of early NSCLC surgery, and the high level of serum TUM2‐PK may be a manifestation of poor prognosis of patients. For patients with poor prognosis and high‐risk early NSCLC, multidisciplinary comprehensive treatment may benefit them, so as to improve the overall cure rate of early NSCLC patients. However, before the serum TUM2‐PK level is included in clinical testing, large sample prospective studies are still needed to further evaluate its application value.

## CONFLICTS OF INTEREST

No conflicts of interest.

## AUTHOR CONTRIBUTION

**Chunhua Xu:** Conceptualization (equal); Writing‐original draft (equal). **Wei Liu:** Investigation (equal); Methodology (equal). **Li Li:** Methodology (equal). **Yuchao Wang:** Formal analysis (equal). **Qi Yuan:** Writing‐original draft (equal); Writing‐review & editing (equal).

## Data Availability

All data analysed are included in this article.
